# Genome-Wide Analysis of Lung Adenocarcinoma Identifies Novel Prognostic Factors and a Prognostic Score

**DOI:** 10.3389/fgene.2019.00493

**Published:** 2019-05-22

**Authors:** Donglai Chen, Yueqiang Song, Fuquan Zhang, Xiaofan Wang, Erjia Zhu, Xi Zhang, Gening Jiang, Siguang Li, Chang Chen, Yongbing Chen

**Affiliations:** ^1^Department of Thoracic Surgery, Shanghai Pulmonary Hospital, Tongji University School of Medicine, Shanghai, China; ^2^Department of Regenerative Medicine, Stem Cell Center, Tongji University School of Medicine, Shanghai, China; ^3^Department of Thoracic Surgery, The Second Affiliated Hospital of Soochow University, Medical College of Soochow University, Suzhou, China

**Keywords:** lung adenocarcinoma, genome-wide, prognostic factor, survival prediction, TCGA

## Abstract

**Background and Objective:**

Lung adenocarcinoma (LUAD) is the most common histological type of all lung cancers and is associated with genetic and epigenetic aberrations. The tumor, node, and metastasis (TNM) stage is the most authoritative indicator of the clinical outcome in LUAD patients in current clinical practice. In this study, we attempted to identify novel genetic and epigenetic modifications and integrate them as a predictor of the prognosis for LUAD, to supplement the TNM stage with additional information.

**Methods:**

A dataset of 445 patients with LUAD was obtained from The Cancer Genome Atlas database. Both genetic and epigenetic aberrations were screened for their prognostic impact on overall survival (OS). A prognostic score (PS) integrating all the candidate prognostic factors was then developed and its prognostic value validated.

**Results:**

A total of two micro-RNAs, two mRNAs and two DNA methylation sites were identified as prognostic factors associated with OS. The low- and high-risk patient groups, divided by their PS level, showed significantly different OS (*p* < 0.001) and recurrence-free survival (RFS; *p* = 0.005). Patients in the early stages (stages I/II) and advanced stages (stages III/IV) of LUAD could be further subdivided by PS into four subgroups. PS remained efficient in stratifying patients into different OS (*p* < 0.001) and RFS (*p* = 0.005) when the low- and high-risk subgroups were in the early stages of the disease. However, there was only a significant difference in OS (*p* = 0.04) but not RFS (*p* = 0.2), between the low-risk and high-risk subgroups when both were in advanced stages.

**Conclusion:**

PS, in combination with the TNM stage, provides additional precision in stratifying patients with significantly different OS and RFS prognoses. Further studies are warranted to assess the efficiency of PS and to explain the effects of the genetic and epigenetic aberrations observed in LUAD.

## Introduction

Lung cancer is the leading cause of global cancer-related mortality, and ranks second in the estimated new cases of cancer in both sexes in the United States (Siegel et al., 2017). Lung adenocarcinoma (LUAD) is the most common histological type of lung cancer, accounting for approximately 50% (Shedden et al., 2008; Warth et al., 2012; Cancer Genome Atlas Research Network, 2014). Currently, the tumor, node, and metastasis (TNM) stage is the most accepted system for estimating the prognosis of patients with LUAD in clinical practice (Warth et al., 2012). However, prognoses of LUAD patients who share the same pathological stage vary considerably (Tsao et al., 2015; Zhang et al., 2015; Liang et al., 2017; Dalwadi et al., 2018). Therefore, a more accurate system is in demand to predict the outcomes of patients with LUAD that can add further valuable information to the TNM stage.

Aberrant genetic and epigenetic modifications of oncogenes and tumor suppressors contribute to the tumorigenesis and progression of LUAD (Khalil et al., 2018; Rowbotham et al., 2018; Tessema et al., 2018; Toyokawa et al., 2018; Wang et al., 2018). Genetic and epigenetic abnormalities have been associated with LUAD patient survival, especially the aberrant expression of cancer-related genes and DNA methylation at specific sites (Uruga et al., 2017; Zhang et al., 2017; Gonzalez-Vallinas et al., 2018; Wang et al., 2018). For instance, using genome-scale DNA methylation profiling, a study identified 164 hypermethylated genes and 57 hypomethylated genes involved in cell differentiation and the epithelial-to-mesenchymal transition in LUAD (Selamat et al., 2012). Notably, DNA methylation also accounts for the alteration of gene expression in LUAD (Zhang et al., 2017; Gao et al., 2018; He et al., 2018), and may thus indirectly affect the biological behaviors and processes of LUAD. Specifically, He et al. (2018) identified an association between aberrant CpG-methylation and the prognostic value of the corresponding gene expression based on 1095 LUAD samples, and identified 10 aberrantly methylated and dysregulated genes with independent prognostic value.

In recent years, a class of small non-coding RNA molecules, called microRNA (miRNA), has been increasingly investigated (Fu et al., 2017; Greenawalt et al., 2018; Othman and Nagoor, 2019). miRNAs can regulate the expression of protein-coding genes by base pairing with the target mRNAs, inducing the degradation or translational repression of the bound mRNAs (Ha and Kim, 2014; Hou et al., 2018). The prognostic significance of miRNAs has also been investigated and confirmed in many studies (Zhang et al., 2017; Gonzalez-Vallinas et al., 2018; Xu et al., 2018). For instance, mir-486 was shown to be a miRNA that is differentially expressed in LUAD and potentially interacts with ITGA11, a cancer-promoting gene (Zhang et al., 2017). Gonzalez-Vallinas et al also reported a significant association between mir-539, mir-323b, and mir-487a upregulation and worse disease-free survival in non-smoker patients with LUAD (Gonzalez-Vallinas et al., 2018).

So far, many studies have established panels of prognostic factors that predict the outcomes of patients with LUAD, based on multiple lines of evidence. However, most studies were conducted without integration of the network constituted by dysregulations at different levels. Because LUAD represents a set of heterogeneous diseases in which aberrations can exist at genome and epigenome levels, we performed a genome-wide analysis, which should provide more comprehensive insight into survival prediction. Using the data of 445 patients from The Cancer Genome Atlas (TCGA) database, we identified prognostic value of two miRNAs, two mRNAs and two methylation sites. A prognostic score (PS) was developed by integrating these factors to stratify LUAD patients with different lengths of survival into subgroups. From our data, combining PS and the TNM stage achieved greater accuracy in predicting the prognoses of patients with LUAD, indicating that PS is a promising system for personalized and precise medicine.

## Materials and Methods

### Data Extraction and Prepossessing

The genome-wide data for 706 LUAD patients was downloaded from TCGA database^1^, including the expression levels of 20530 mRNAs, 2228 miRNAs and 485577 DNA methylation sites, together with the outcomes of 630 patients. The exclusion criteria were listed as follows: (1) Patients whose genomic or epigenomic information was absent; (2) Genes lacking information on either their transcript (mRNA or miRNA) or DNA methylation levels in more than half the LUAD samples; (3) Patients whose survival information was unavailable. Ultimately, a total of 445 LUAD patients were included in the study, together with 16928 mRNAs, 453 miRNAs and 395963 DNA methylation sites.

### Identification of Survival-Associated Transcripts and DNA Methylation Sites

A Cox regression model was used to evaluate the association of gene transcripts (mRNA or miRNA) and DNA methylation sites with lengths of overall survival (OS). A univariate Cox regression analysis was initially used, followed by the screening of included potential factors with a *p* ≤ 0.1 for further analysis.

Afterward, considering the remaining large numbers of gene transcripts and methylated sites, we performed a Lasso-Cox analysis to screen and shrink the data. We then used multivariate Cox regression to further analyze the association between the gene transcripts or DNA methylation sites with OS, while adjusting for other clinicopathological factors.

### Identifying and Screening Potential miRNA Targets

We retrieved the potential target genes of miRNAs that had already been shown to be significantly associated with OS from miRTarBase (the experimentally validated microRNA-target interactions database, release 7.0) (Hou et al., 2018). Lasso-Cox regression was then used to screen the mRNAs of genes which were identified as targets of miRNAs with high-confidence (*p* ≤ 0.05).

### Calculation of Spearman’s Correlation Coefficients

The direction of association among the transcripts and methylation sites were calculated with Spearman’s correlation in the 445 LUAD tissues. If an mRNA tended to increase when miRNA or methylation increased, the Spearman’s correlation coefficient was positive. If an mRNA tended to decrease when miRNA or methylation increased, the Spearman’s correlation coefficient was negative. We set a threshold of 0 with which to assess the candidate miRNAs, mRNAs and methylation sites. Any pair with a correlation coefficient value < 0 was considered to be negatively correlated, whereas any pair with a correlation coefficient value > 0 as positively correlated.

### External Validation of Identified Transcripts and Methylation Sites

We validated the prognostic value of our candidate transcripts in KM-Plot^2^. The impact of the candidate methylation sites on survival was confirmed in MethSurv^3^ (Modhukur et al., 2018). We used Jetset to select the corresponding probe sets for the candidate mRNAs and miRNAs because a given gene may be detected by multiple probe sets, which may lead to inconsistent or even contradictory measurements (Li et al., 2011).

### Construction and Validation of PS

To further assess the predictive ability of the significant factors identified, we constructed a PS as an integrated predictor. PS was calculated as a weighted sum of the expression levels of the transcripts and DNA methylation sites present in a given sample (Hou et al., 2018). For specimen i the calculation formula for PS was shown as follows:

$$PS=\overset{n}{\underset{i=1}{\Sigma }}\beta ixi$$

The weight of each variable is represented by the Cox regression coefficient β, and the expression level is denoted by x. A greater value of PS indicates a worse prognosis.

We divided the patients into either the high-risk or low-risk group according to the median value of PS. Each group was subdivided into the early-stage (stages I–II) and advanced-stage (stages III–IV) subgroups based on the pathological stage. The Kaplan–Meier method and log-rank tests were used to assess the differences in OS and RFS between in the subgroups.

### Statistical Analysis

All statistical analyses were performed with R version 3.4.4 (packages glmnet_2.0-16, survival_2.4-3; Institute for Statistics and Mathematics, Vienna, Austria). A two-tailed *p* < 0.05 was considered statistically significant.

## Results

### General Information on Patients With LUAD

The clinicopathological characteristics of the LUAD patients in our study are shown in Table 1. Of the 445 patients, 210 (47.2%) were male and 235 (52.8%) were female. The median age was 66 years (ranging from 39 to 88). Patients with early-stage LUAD constituted the majority of our cohort. The primary tumor mainly was mainly located in the upper lobe on either side.

**Table 1:** Distributions of the demographic and clinical variables of 445 patients with lung adenocarcinoma patients.

Characteristic	Number (range)
Age at first diagnosis (median, range)	66 (39–88)
Gender	
Male	210
Female	235
Pathology (Histologic subtypes)	
Lepidic-predominant Adc	10
Acinar-predominant Adc	59
Papillary-predominant Adc	20
Micropapillary-predominant Adc	21
Solid-predominant Adc	34
Invasive mucinous Adc	7
Pathological stages	
Stage I	239
Stage II	109
Stage III	72
Stage IV	20
Unknown	5
Smoking history	
Lifelong non-smoker	65
Smoker	359
Current smoker	103
Current reformed smoker for < or = 15 years	145
Current reformed smoker for > 15 years	111
Number of packs smoked (N) per year	
N = 0	65
0 < N ≤ 20	112
20 < N ≤ 40	140
40 < N ≤ 60	113
60 < N ≤ 80	29
N > 80	38
Additional pharmaceutical therapy	
Yes	54
No	72
Additional radiation therapy	
Yes	65
No	63
Targeted molecular therapy	
Yes	138
No	247
Location	
RUL	159
RML	19
RLL	83
LUL	103
LLL	69


*Adc, adenocarcinoma; RUL, right upper lobe; RML, right middle lobe; RLL, right lower lobe; LUL, left upper lobe; LLL, left lower lobe.*

**FIGURE 1:**
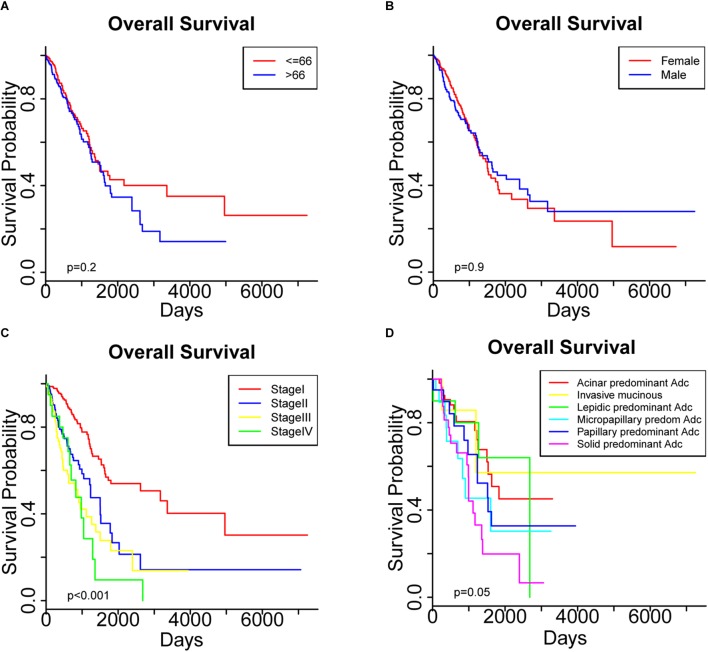
Overall survival was compared among patients stratified by **(A)** age, **(B)** sex, **(C)** pathological stage and **(D)** histologic subtypes.

As shown in Table 2, we examined the association between each clinicopathological characteristic and OS. A univariate Cox regression analysis indicated that a higher TNM stage was significantly associated with poorer OS (Table 2). Meanwhile, a Kaplan-Meier survival analysis showed significantly different OS among the patients with different TNM stages (Figure 1C), but not among those who differed in age or sex (Figure 1A,B). Interestingly, a trend toward different OS among patients with different histologic subtypes was observed, but the *p* value was only marginally significant (Figure 1D). In the multivariate regression analysis, only a higher TNM stage remained a significant risk factor for OS (Table 2).

**Table 2:** Risk factors in the patient cohort in our study.

	Univariate analysis			Multivariate analysis		
Characteristics	HR	95%CI	*p-*value	HR	95%CI	*p-*value
Age						
≤ 65						
> 66	1.233	0.900–1.688	0.192	1.153	0.652–2.041	0.624
Gender						
Female						
Male	1.016	0.744–1.386	0.922	1.259	0.712–2.226	0.428
Histologic subtypes						
Acinar predominant Adc						
Lepidic predominant Adc	1.067	0.356–3.197	0.907	0.782	0.233–2.625	0.69
Micropapillary predominant Adc	2.015	0.890–4.566	0.093	1.365	0.594–3.135	0.464
Papillary predominant Adc	1.48	0.685–3.197	0.318	0.899	0.397–2.036	0.799
Solid predominant Adc	2.66	1.375–5.148	0.004	1.752	0.855–3.593	0.126
Invasive mucinous Adc	0.81	0.186–3.527	0.779	0.709	0.156–3.223	0.656
Pathological stage						
Stage I						
Stage II	2.371	1.609–3.493	0	2.569	1.332–4.956	0.005
Stage III	3.419	2.280–5.128	0	2.16	1.060–4.400	0.034
Stage IV	3.863	2.146–6.951	0	2.139	0.715–6.403	0.174
T stage						
T1						
T2	1.42	0.978–2.060	0.065	NA	NA	NA
T3	2.411	1.341–4.333	0.003	NA	NA	NA
T4	2.727	1.396–5.330	0.003	NA	NA	NA
N stage						
N0						
N1	2.184	1.502–3.176	0	NA	NA	NA
N2	3.111	2.096–4.619	0	NA	NA	NA
M stage						
M0						
M1	2.155	1.208–3.845	0.009	NA	NA	NA


*Adc, adenocarcinoma; HR, hazard ratio; CI, confidence interval; NA, not available.*

### Identification of Transcripts and DNA Methylation Sites as Prognostic Factors

From 16928 mRNAs, 453 miRNA and 395963 DNA methylation sites, a total of 26 miRNAs, 15 mRNAs and 11 DNA methylation sites were initially identified as factors associated with OS using univariate Cox and Lasso-Cox analyses (Supplementary Table S1). Next, a list of 2882 genes was then retrieved from the miRTarBase database, of which 21 were identified as high-confidence (*p* ≤ 0.05) targets of the 26 survival-associated miRNAs. A Lasso-Cox analysis was used to select the mRNAs of the 21 genes that interacted with the corresponding survival-related miRNAs. After a multivariate Cox regression analysis of the 73 potential prognostic factors, the overexpression of two miRNAs (MIMAT0002890 and MIMAT0000426) and the hypermethylation of two sites (cg12141052 and cg16404170) were confirmed as significant predictors of worse prognosis (Table 3), and the higher expression level of two mRNAs (CDADC1, FAHD2B) was significantly associated with a better prognosis (Table 3). Therefore, the final list of candidate prognostic factors for LUAD contained 6 biomarkers, including two miRNAs, two mRNAs and two methylation sites.

**Table 3:** Genome-wide prognostic factors identified in our study.

Molecular	Name	Coefficient	HR	95% CI	SE	z-value	*p-*value
miRNA	MIMAT0002890	0.358	1.431	1.191, 1.718	0.094	3.829	0
	MIMAT0000426	0.246	1.278	0.999, 1.635	0.126	1.954	0.051
mRNA	CDADC1	-0.578	0.561	0.387, 0.814	0.19	-3.047	0.002
	FAHD2B	-0.276	0.759	0.643, 0.895	0.084	-3.281	0.001
Methylation site	cg12141052	4.045	57.117	8.653, 377.015	0.963	4.201	0
	cg16404170	3.495	32.959	6.593, 164.761	0.821	4.257	0


*HR, hazard ratio; CI, confidence interval; SE, standard error; z value, Wald z-statistic value.*

The Spearman’s rank correlation coefficients for these candidate transcripts and methylation levels were then calculated for the LUAD cohort of 445 patients (Table 4 and Supplementary Figure S1).

**Table 4:** Spearman’s correlation coefficients among the prognostic factors identified in the study.

Factors	MIMAT0002890	MIMAT0000426	CDADC1	FAHD2B	cg12141052	cg16404170
MIMAT0002890	1.000	0.245	-0.004	-0.028	-0.119	0.136
MIMAT0000426	0.245	1.000	-0.042	0.000	-0.029	0.145
CDADC1	-0.004	-0.042	1.000	0.174	-0.160	-0.165
FAHD2B	-0.028	0.000	0.174	1.000	-0.053	-0.143
cg12141052	-0.119	-0.029	-0.160	-0.053	1.000	0.021
cg16404170	0.136	0.145	-0.165	-0.143	0.021	1.000


### External Validation of Candidate Transcripts and Methylation Sites

As shown in Supplementary Figures S2–S4, a univariate Cox proportional hazards regression analysis showed that the six candidate factors identified from either genome or epigenome of LUAD were significantly associated with the survival of patient cohorts in other databases. Moreover, the relationships between their expression levels and the survival rate of LUAD patients were consistent with our findings.

### Validation of the Integrated Prognostic Factors

**FIGURE 2:**
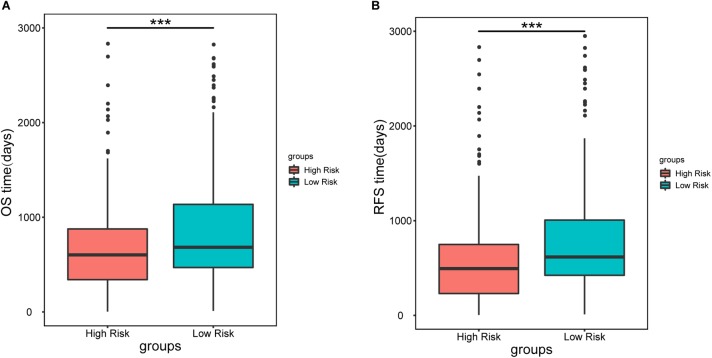
Median overall survival **(A)** and recurrence-free survival **(B)** in patients in the high-risk (PS > 1.88) and low-risk groups (PS < 1.88). Patients in the high-risk group showed a significantly shorter survival than those in the low-risk group (^∗∗∗^*p* < 0.001).

**FIGURE 3:**
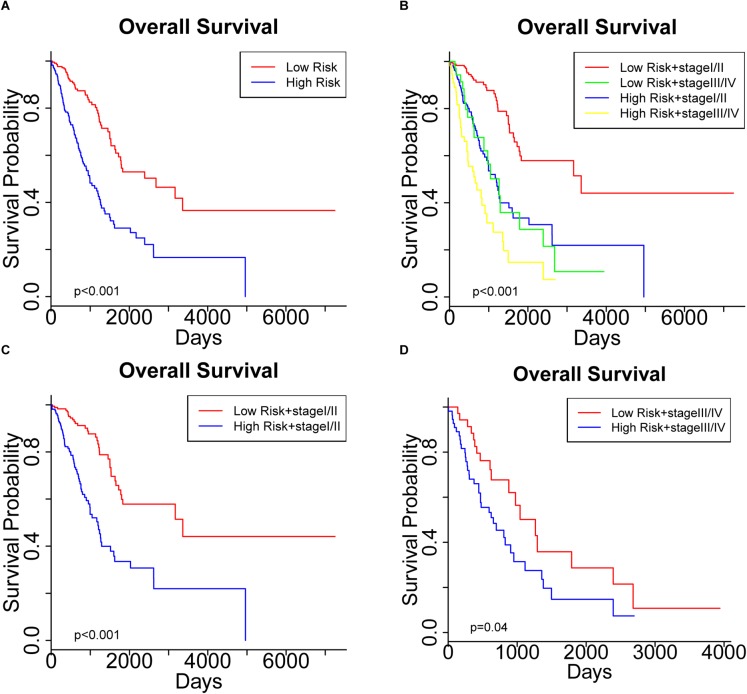
Kaplan Meier curve showing the overall survival (OS) of the patient cohort grouped by **(A)** prognostic score (PS), and **(B)** PS plus pathological stage. OS of patients stratified by PS in subgroups with **(C)** early-stage tumors and **(D)** advanced- stage tumors.

**FIGURE 4:**
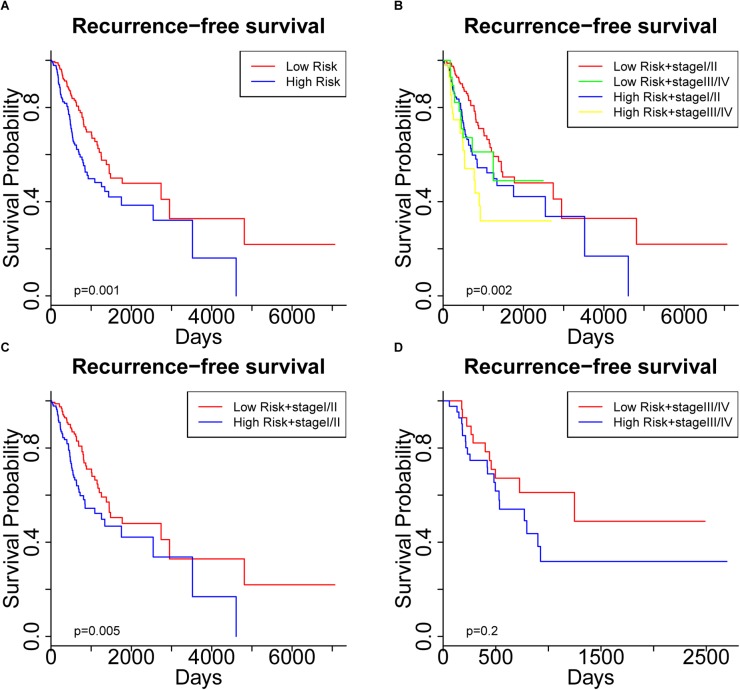
Kaplan Meier curve showing recurrence-free survival (RFS) of the patient cohort grouped by **(A)** prognostic score (PS), and **(B)** PS plus pathological stage. RFS of the patients stratified by PS in subgroups with **(C)** early-stage tumors and **(D)** advanced-stage tumors.

To further assess the predictive capacity of all the candidate prognostic factors, PS was established as an integrated prognostic predictor. To verify the efficiency of PS, the 445 LUAD patients were divided into two groups stratified by the median PS. The high-risk group (PS > 1.88) included 223 patients and the low-risk group (PS < 1.88) included 222 patients. As shown in Figure 2, the patients in the low- and high-risk groups displayed significantly different median OS (1070.8 vs. 753.9 days, *p* < 0.001) and RFS (900.8 vs. 668.2, *p* = 0.005). As shown in Figure 3A, 4A, the Kaplan-Meier curves and log-rank tests indicated significant differences in the OS [hazard ratio (HR): 2.861, 95% confidence interval (CI): 2.052–3.988, *p* < 0.001] and RFS (HR: 1.77, 95% CI: 1.255–2.497, *p* = 0.001) between two groups.

Significantly different OS and RFS were also observed among the subgroups in further analyses (Figure 3B, 4B). On the one hand, PS remained efficient in stratifying the patients into different OS (HR: 3.177, 95%CI: 2.110–4.783, *p* < 0.001) and RFS (HR: 1.752, 95% CI: 1.184–2.595, *p* = 0.005) when the low-risk and high-risk subgroups were in the early stages of the disease (Figure 3C, 4C). However, there was only a significant difference in OS (HR: 1.806, 95% CI: 1.019–3.200, *p* = 0.04) but not RFS (HR: 1.594, 95% CI: 0.763–3.333, *p* = 0.2) between the two subgroups when both were in the advanced stages of the disease (Figure 3D, 4D). On the other hand, the pathological stage could distinguish significantly different OS (low-risk group: HR: 3.341, 95% CI: 1.888–5.912, *p* < 0.001; high-risk group: HR: 1.955, 95% CI: 1.305–2.929, *p* < 0.001) but not RFS (low-risk group: HR: 1.472, 95% CI: 0.878–2.467, *p* = 0.1; high-risk group: HR: 1.604, 95% CI: 0.829–3.104, *p* = 0.2) in the low-risk and high-risk groups. Thus, PS was proved to be a useful prognostic indicator that can supplement additional information to the TNM stage, especially for LUAD patients in the early stages of the disease. Our study suggests that the combination of the TNM stage and PS increases the accuracy in predicting the outcomes of patients with LUAD.

## Discussion

The past decade has witnessed rapid progress in next-generation sequencing and its increasing application in preclinical practice. In recent years, several studies have attempted to associate the transcriptome or epigenome with the clinical outcomes of patients with LUAD (Selamat et al., 2012; Zhang et al., 2017; Gao et al., 2018; He et al., 2018). Zhang et al. analyzed and validated the expression profiles and prognostic values of the mRNAs of five differentially expressed genes associated with DNA methylation in LUAD (Zhang et al., 2017), increasing the likelihood that altered signature genes will become useful biomarkers. Using a TCGA dataset, He et al. (2018) disentangled the relationships between aberrant CpG-methylation and gene expression to identify 10 aberrantly methylated and dysregulated genes. However, their study only focused on the ability of individual genes to predict OS. Another TCGA-based study examined the feasibility of integrating prognosis-related methylation-driven genes into a risk model to predict the OS of patients with LUAD, which also involved a joint survival analysis based on methylation sites and gene expression (Gao et al., 2018). Nevertheless, it remained unclear whether a risk model could improve the accuracy of the TNM stage for survival estimation. Furthermore, no information was given on the predictive value of their method in distinguishing RFS in LUAD patients. None of these studies included the histologic subtypes proposed by the International Association for the Study of Lung Cancer/American Thoracic Society/European Respiratory Society (IASLC/ATS/ERS) (Travis et al., 2011; Warth et al., 2012; Hung et al., 2014) as an independent prognostic factor.

To the best of our knowledge, this is the first study to integrate genetic and epigenetic modifications for survival prediction in LUAD patients using TCGA samples. With a comprehensive analysis and screening of mRNA expression, miRNAs and DNA methylation sites based on samples from 445 patients, we identified a set of prognostic factors from both the transcriptome and epigenome. Notably, we included the histologic subtypes and the TNM stages in our initial survival analysis. In this way, we developed a novel subgrouping system that integrates PS and the TNM stage to predict the survival of patients with LUAD.

We started by identifying the clinicopathological characteristics associated with the OS of patients with LUAD. Both Cox regression and Kaplan-Meier survival analyses confirmed the significant prognostic impact of the TNM stage (Table 2 and Figure 1). Further screening of genetic and epigenetic aberrations identified a collection of 26 miRNAs, 15 mRNAs and 11 DNA methylation sites whose expression or methylation levels were significantly associated with OS (Supplementary Table S1). Since miRNAs exert their function by regulating the expression of their target mRNAs, we retrieved the potential targets of these 26 miRNAs and performed a LASSO-Cox analysis to select 21 mRNAs as high-confidence miRNA targets. This provided clues to the potential molecular interactions by which these miRNAs affect the clinical outcomes. Considering the interactions between these candidate prognostic factors into consideration, we performed a multivariate Cox regression to finally identify a list of six survival-related biomarkers. From our data, the expression levels of two mRNAs (CDADC1 and FAHD2B), two miRNAs (MIMAT0002890 and MIMAT0000426) and methylation of two sites (cg12141052 and cg16404170) were strongly associated with the clinical outcomes. PS was then computed as a predictor that integrated these candidate biomarkers and stratified the patients into low-risk (PS < 1.88) and high-risk groups (PS > 1.88). The efficiency of PS was confirmed by our success in distinguishing the OS and RFS of LUAD patients (Figure 3, 4). A subgroup analysis further demonstrated that a more precise prediction of survival could be achieved for patients with LUAD by combining PS with the TNM stage, which should allow more timely therapeutic interventions.

To be noted, Targetscan^4^ was preferentially considered for the validation of our candidate miRNAs, however, the small number of miRNA targets shared between miRTarBase and Targetscan limited its use (Supplementary Figure S5).

Ten survival-associated genes, whose aberrant expression was affected by methylation, have been identified previously by He et al. (2018) from the TCGA data portal^5^. Therefore we attempted to include the mRNAs of these 10 genes in our transcripts for further screening. However, as shown in Supplementary Table S2 and Supplementary Figures S6–S8, integrating the mRNA of BLK which was identified in the Cox regression analysis into PS did not improve its predictive ability.

In terms of the disproportionate number of non-smokers in the selected patient cohort, secondary analyses were therefore performed to assess the potential value of PS in predicting the survival of the non-smokers and smokers in our cohort. As shown in Supplementary Figure S9A, Kaplan-Meier curves and the log-rank test indicated a significant difference in OS between two groups (HR: 2.785, 95% CI: 1.071–7.24, *p* = 0.03). Significantly different OS was also observed among subgroups stratified by PS plus the TNM stage (Supplementary Figure S9B). However, the performance of PS was not satisfactory for the non-smokers (Supplementary Figures S9C,D), especially in stratifying patients with advanced-stage LUAD into subgroups with different OS, which might be attributed to the limited number of non-smokers (*n* = 65). On the contrary, PS remained consistently efficient in stratifying OS in the smokers (*n* = 359) (Supplementary Figure S10).

There were several limitations to our study. For instance, risk factors such as packages of cigarettes and adjuvant therapy were not included in our analysis because of their interpatient heterogeneity. Moreover, the histologic subtypes was unsatisfactory in distinguishing prognoses in the multivariate analysis which could be explained by the missing histologic information for almost half the patients. It is noteworthy that the failure of PS to distinguish RFS in the advanced-stage subgroups (*p* = 0.2) should be possibly attributed to the limited number of LUAD patients with advanced-stage disease. Last but not least, the clinical utility of PS identified here may be limited in patients with small-sized lesions because of the difficulty in extracting sufficient RNA and protein. More studies are warranted to assess the roles of these candidate prognostic factors in LUAD.

## Conclusion

In conclusion, using a TCGA dataset of 445 LUAD patients, we identified six prognostic factors (two mRNAs, two miRNAs and two DNA methylation sites) for LUAD from the genome and epigenome, and developed PS from them. Combining the TNM stage and PS provided additional precision in stratifying patients into significantly different OS and RFS subgroups. Further studies are warranted to assess the efficiency of PS and to explain the effects of these observed genetic and epigenetic aberrations in LUAD.

## Author Contributions

DC, YS, SL, CC, and YC designed the study. DC, YS, FZ, and EZ performed the data analysis. DC, YS, FZ, XW, and XZ wrote the manuscript. GJ, SL, CC, and YC reviewed and edited the manuscript. YC and FZ afforded to conduct our study.

## Conflict of Interest Statement

The authors declare that the research was conducted in the absence of any commercial or financial relationships that could be construed as a potential conflict of interest.

## Footnotes

1 https://xenabrowser.net

2 http://kmplot.com/analysis/index.php?p=background

3 https://biit.cs.ut.ee/methsurv/

4 http://www.targetscan.org/vert_72/

5 https://cancergenome.nih.gov

## Supplementary Material

Figure S1 Plot of Spearman’s rank correlation coefficients among candidate transcripts and methylation levels in LUAD (*n* = 445).

Figure S2 Differential expression and prognostic impact of **(A)** FADH2B and **(B)** CDADC1 (two candidate mRNAs) in LUAD patients. Kaplan-Meier curves of 720 LUAD patients, who were separated into high-expression and low-expression groups, using as cutoffs the best-performing thresholds of the different genes. All values were significant (*p* < 0.05).

Figure S3 Differential expression and prognostic impact of **(A)** MIMAT0002890 and **(B)** MIMAT0000426 (two candidate miRNAs) in LUAD patients. Kaplan-Meier curves of 720 LUAD patients, who were separated into high-expression and low-expression groups, using as cutoffs the best-performing thresholds of the different miRNAs. All values were significant (*p* < 0.05).

Figure S4 Differential expression and prognostic impact of **(A)** cg12141052 and **(B)** 16404170 (two candidate methylation sites) in LUAD patients. Kaplan-Meier curves of 720 LUAD patients, who were separated into high-expression and low-expression groups, using as cutoffs the best-performing thresholds. All values were significant (*p* < 0.05).

Figure S5 Venn diagrams representing 1161 miRNA targets that overlapped between miRTarBase and Targetscan.

Figure S6 Median overall survival **(A)** and recurrence-free survival **(B)** of patients in the high-risk (PS > 2.78) and low-risk groups (PS < 2.78). Patients in the high-risk group showed a significantly shorter survival than those in the low-risk group (^∗∗∗^*p* < 0.001).

Figure S7 Kaplan Meier curve showing the overall survival (OS) of the patient cohort grouped by **(A)** recombinant prognostic score (PS), and **(B)** recombinant PS plus pathological stage. OS of the patients stratified by PS in subgroups with **(C)** early-stage disease and **(D)** advanced-stage disease.

Figure S8 Kaplan Meier curve showing recurrence-free survival (RFS) of the patient cohort grouped by **(A)** recombinant prognostic score (PS), and **(B)** recombinant PS plus pathological stage. RFS of the patients stratified by PS in subgroups with **(C)** early-stage disease and **(D)** advanced-stage disease.

Figure S9 Kaplan Meier curve showing the overall survival (OS) of the non-smokers in our cohort grouped by **(A)** prognostic score (PS), and **(B)** PS plus pathological stage. OS of the patients stratified by PS in subgroups with **(C)** early-stage disease and **(D)** advanced-stage disease.

Figure S10 Kaplan Meier curve showing the overall survival (OS) of the smokers in our cohort grouped by **(A)** prognostic score (PS), and **(B)** PS plus pathological stage. OS of the patients stratified by PS in subgroups with **(C)** early-stage disease and **(D)** advanced-stage disease.

Table S1 Transcripts and DNA methylation sites whose expression levels showed significant association with overall survival.

Table S2 Integrated genome-wide prognostic factors in our study.
