# Supplementary figures and images for: Genome-Wide Analysis of Lung Adenocarcinoma Identifies Novel Prognostic Factors and a Prognostic Score
Source: Front Genet. 2019 May 22;10:493. doi: 10.3389/fgene.2019.00493 (PMC6539224; doi:10.3389/fgene.2019.00493)

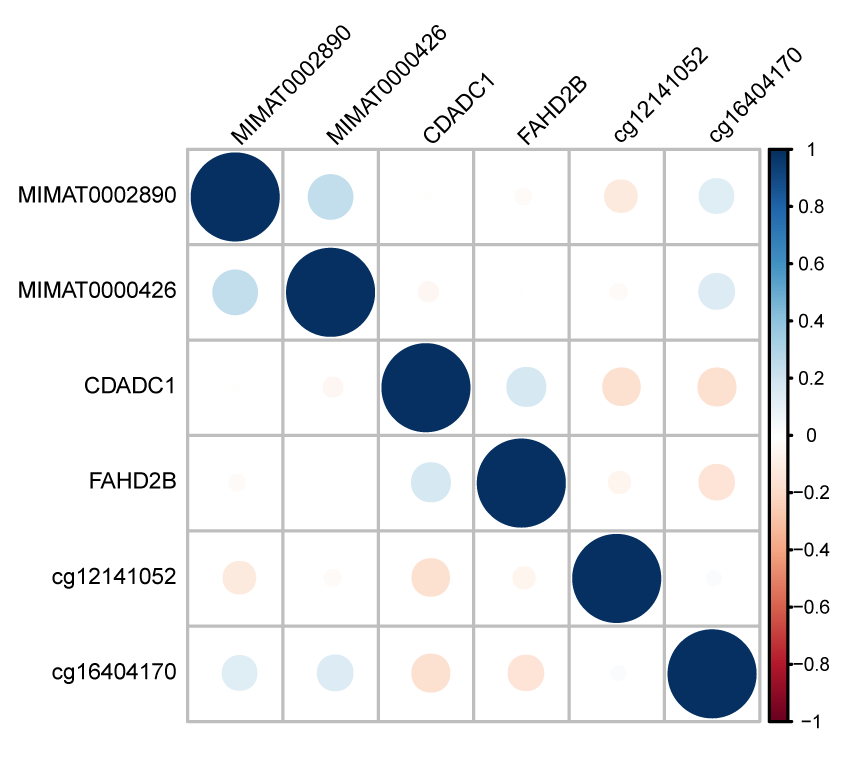

Supplement: Figure S1 — Plot of Spearman’s rank correlation coefficients among candidate transcripts and methylation levels in LUAD (n = 445). [file Image_1.TIF]

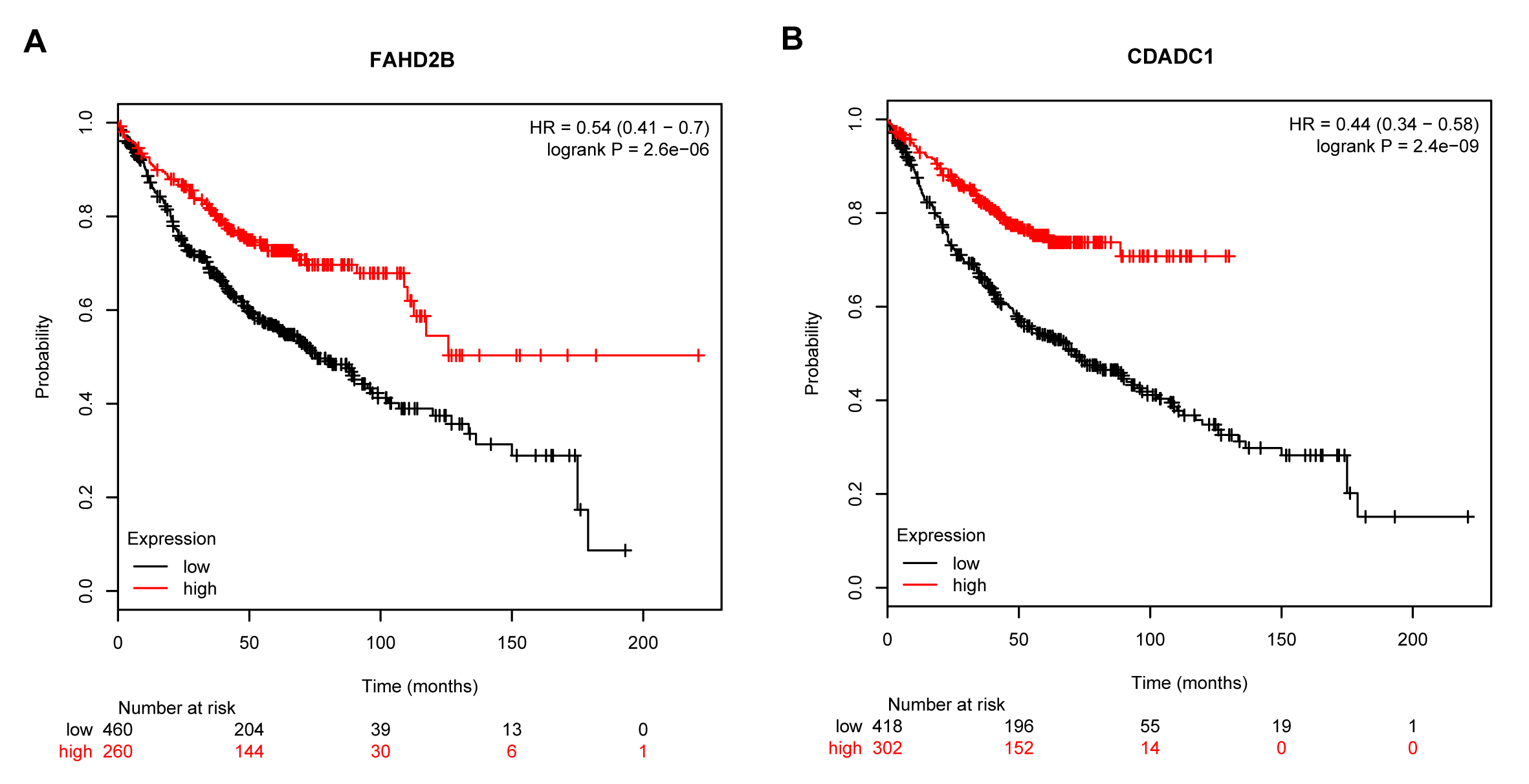

Supplement: Figure S2 — Differential expression and prognostic impact of (A) FADH2B and (B) CDADC1 (two candidate mRNAs) in LUAD patients. Kaplan-Meier curves of 720 LUAD patients, who were separated into high-expression and low-expression groups, using as cutoffs the best-performing thresholds of the different genes. All values were significant (p < 0.05). [file Image_2.TIF]

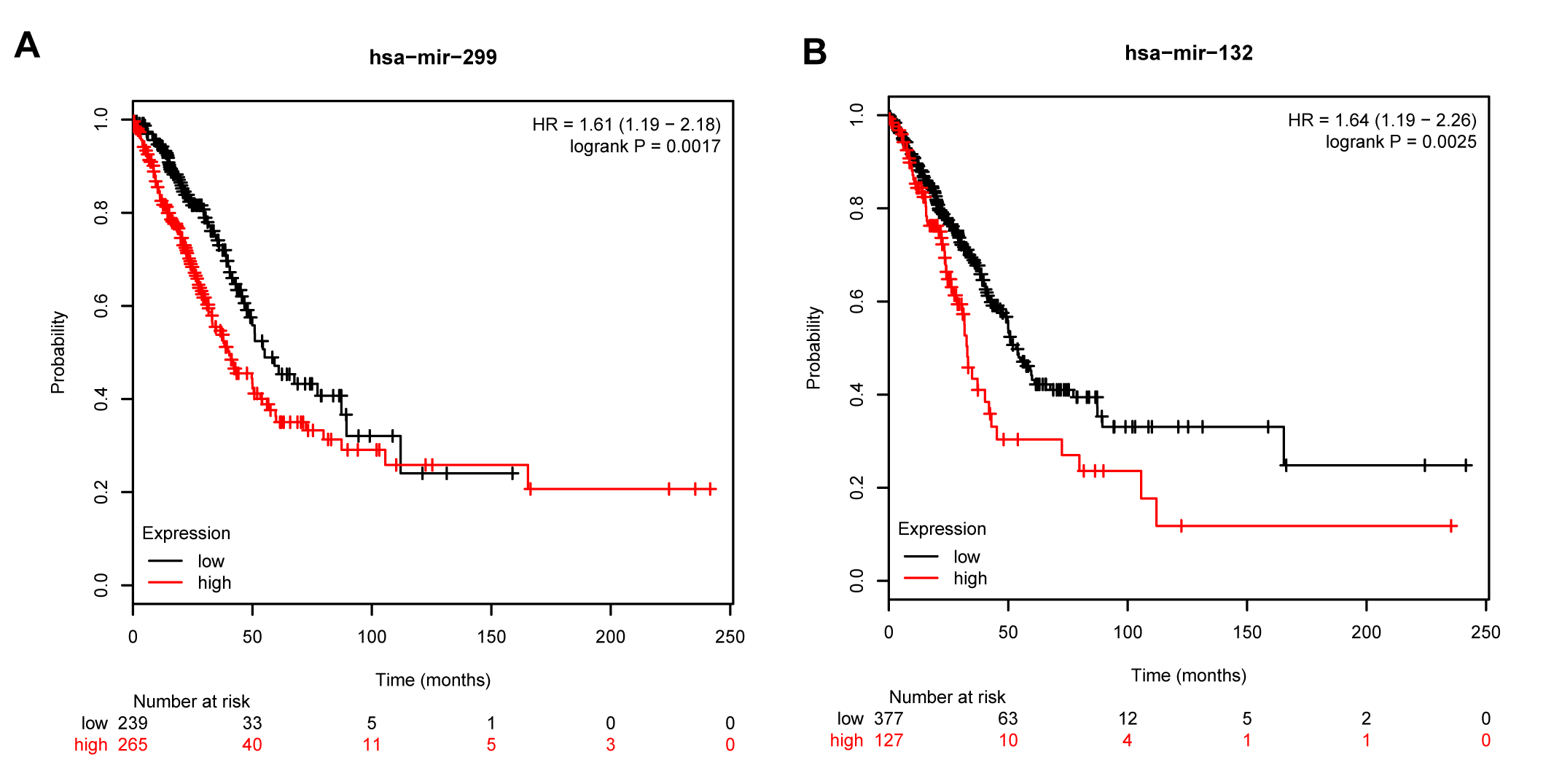

Supplement: Figure S3 — Differential expression and prognostic impact of (A) MIMAT0002890 and (B) MIMAT0000426 (two candidate miRNAs) in LUAD patients. Kaplan-Meier curves of 720 LUAD patients, who were separated into high-expression and low-expression groups, using as cutoffs the best-performing thresholds of the different miRNAs. All values were significant (p < 0.05). [file Image_3.TIF]

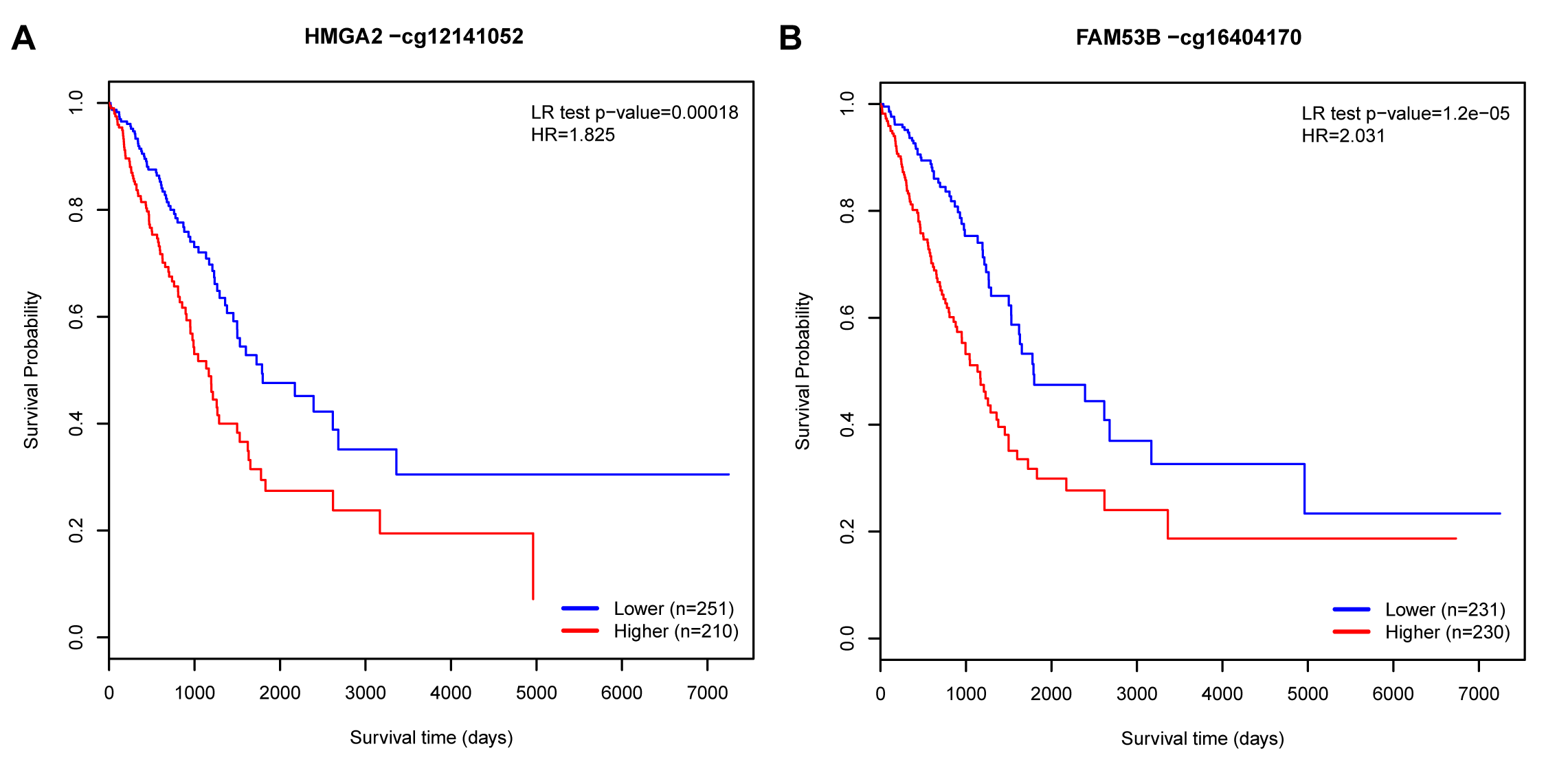

Supplement: Figure S4 — Differential expression and prognostic impact of (A) cg12141052 and (B) 16404170 (two candidate methylation sites) in LUAD patients. Kaplan-Meier curves of 720 LUAD patients, who were separated into high-expression and low-expression groups, using as cutoffs the best-performing thresholds. All values were significant (p < 0.05). [file Image_4.TIF]

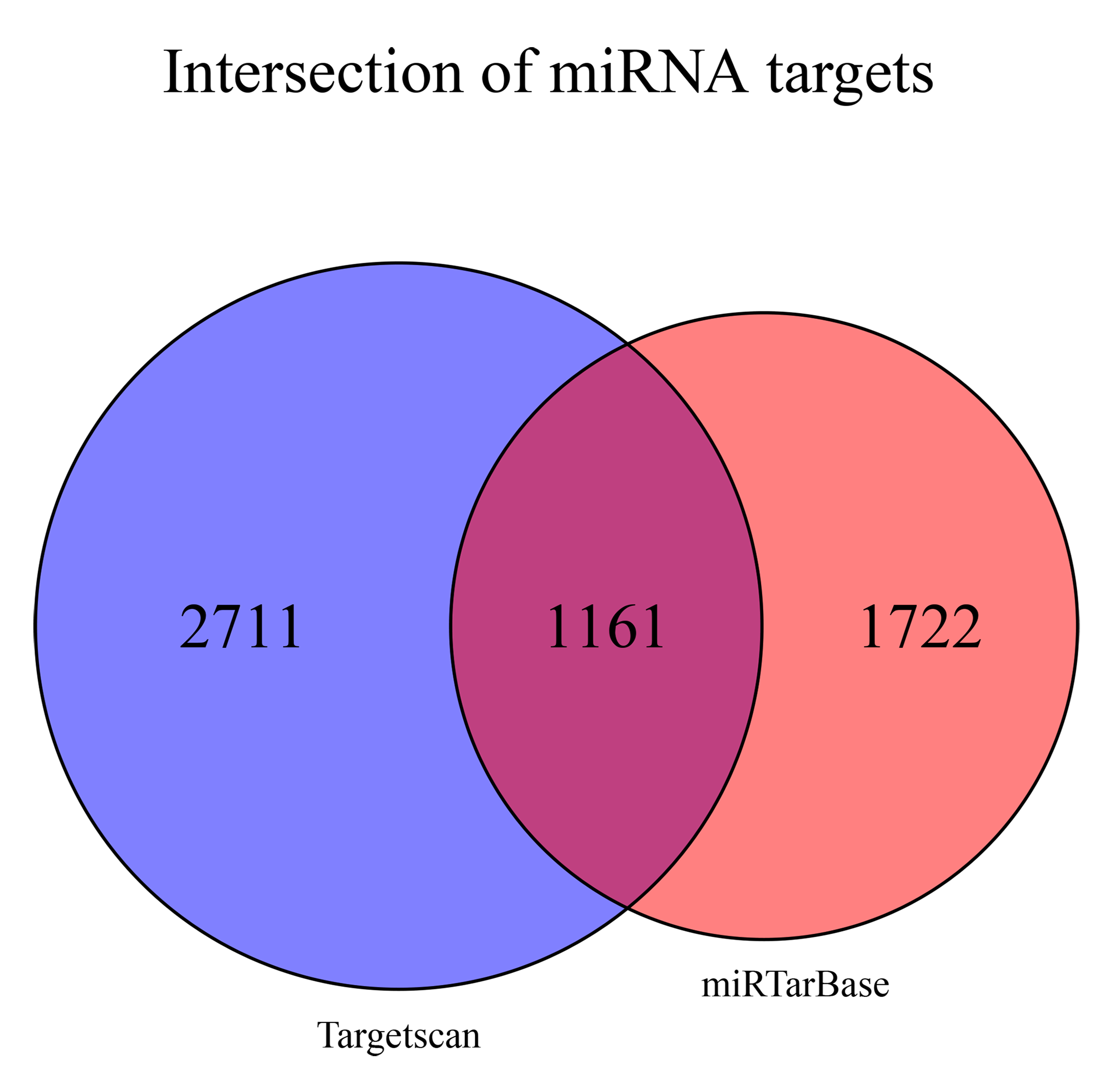

Supplement: Figure S5 — Venn diagrams representing 1161 miRNA targets that overlapped between miRTarBase and Targetscan. [file Image_5.TIF]

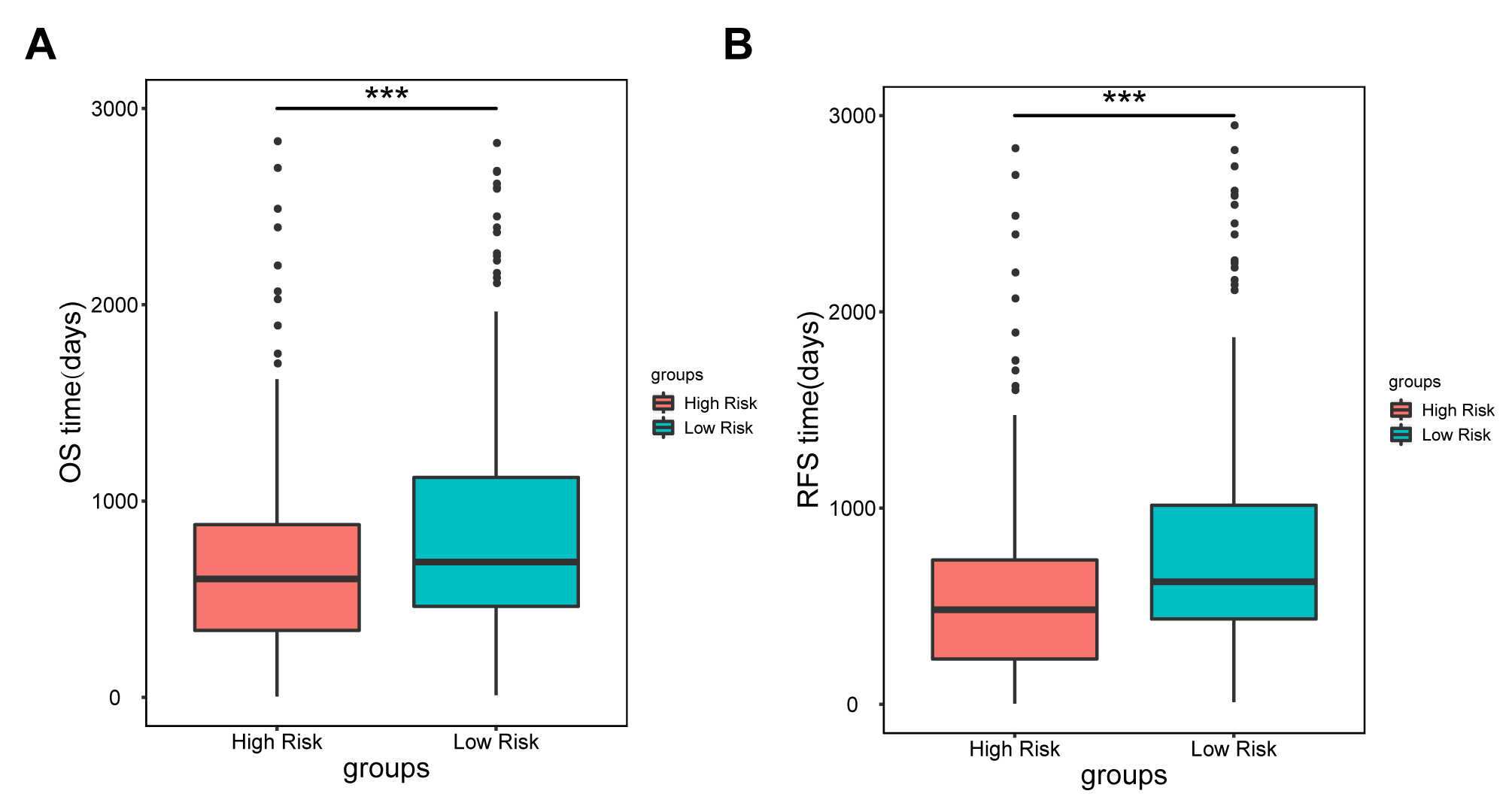

Supplement: Figure S6 — Median overall survival (A) and recurrence-free survival (B) of patients in the high-risk (PS > 2.78) and low-risk groups (PS < 2.78). Patients in the high-risk group showed a significantly shorter survival than those in the low-risk group (∗∗∗p < 0.001). [file Image_6.TIF]

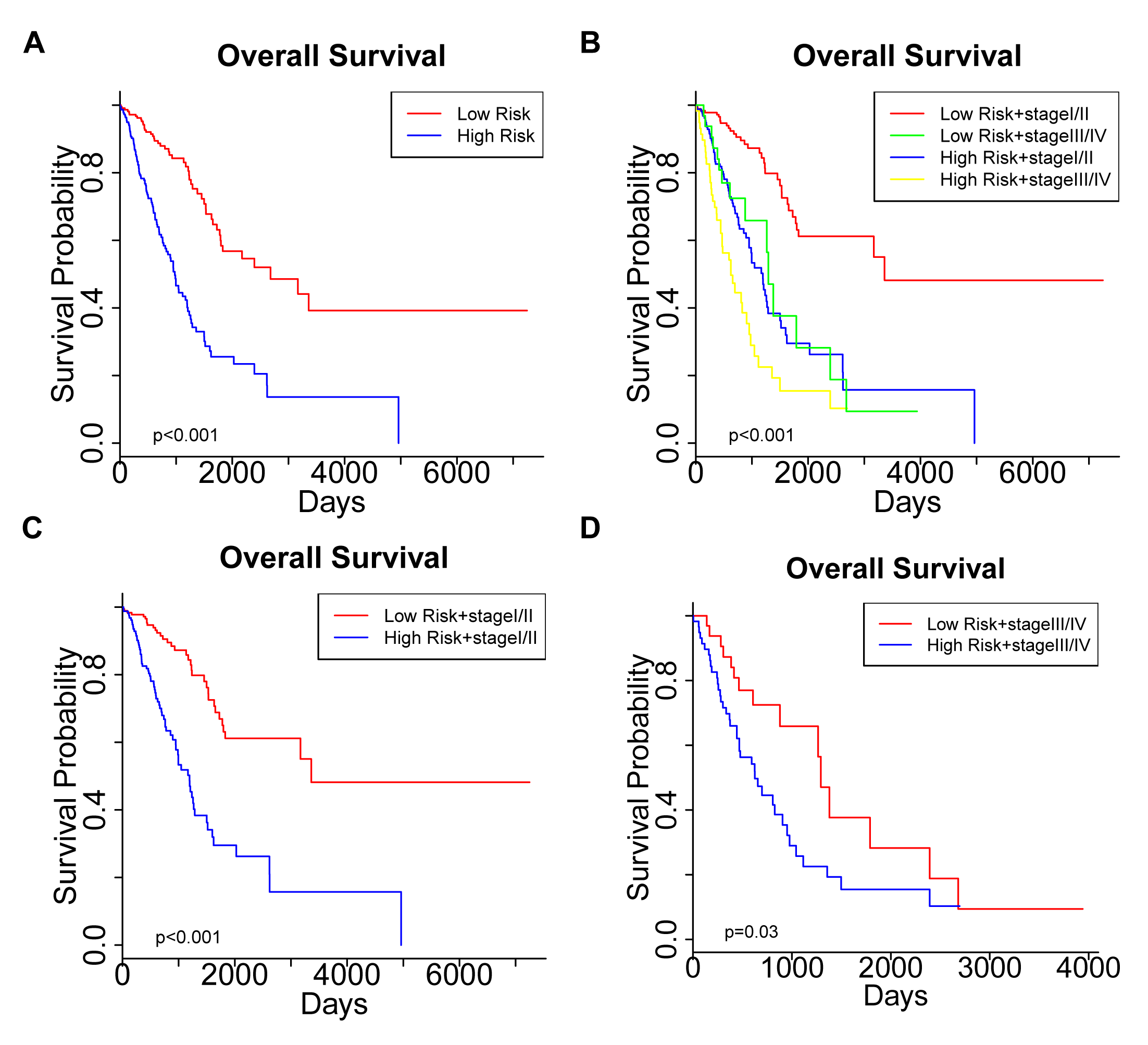

Supplement: Figure S7 — Kaplan Meier curve showing the overall survival (OS) of the patient cohort grouped by (A) recombinant prognostic score (PS), and (B) recombinant PS plus pathological stage. OS of the patients stratified by PS in subgroups with (C) early-stage disease and (D) advanced-stage disease. [file Image_7.TIF]

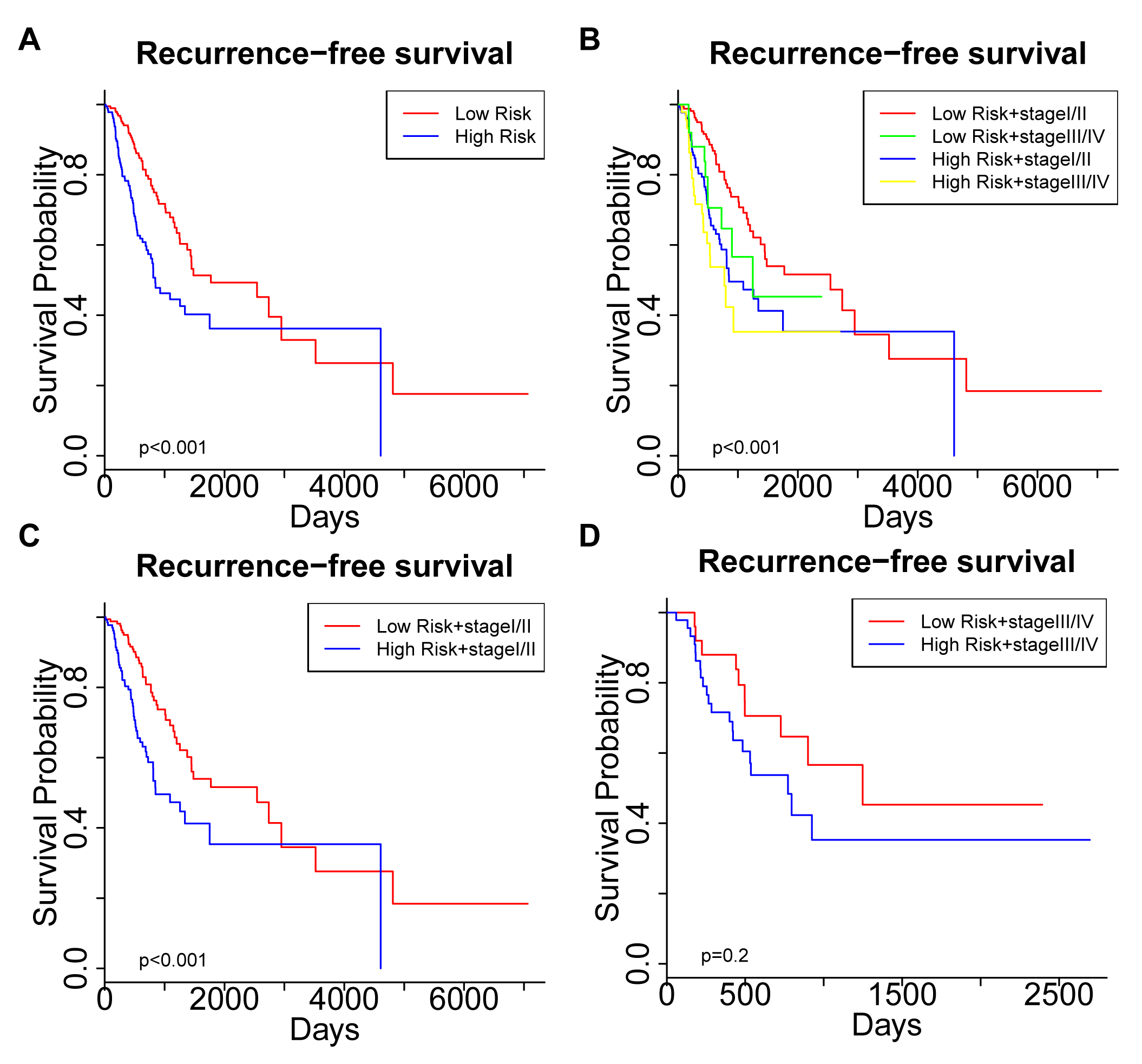

Supplement: Figure S8 — Kaplan Meier curve showing recurrence-free survival (RFS) of the patient cohort grouped by (A) recombinant prognostic score (PS), and (B) recombinant PS plus pathological stage. RFS of the patients stratified by PS in subgroups with (C) early-stage disease and (D) advanced-stage disease. [file Image_8.TIF]

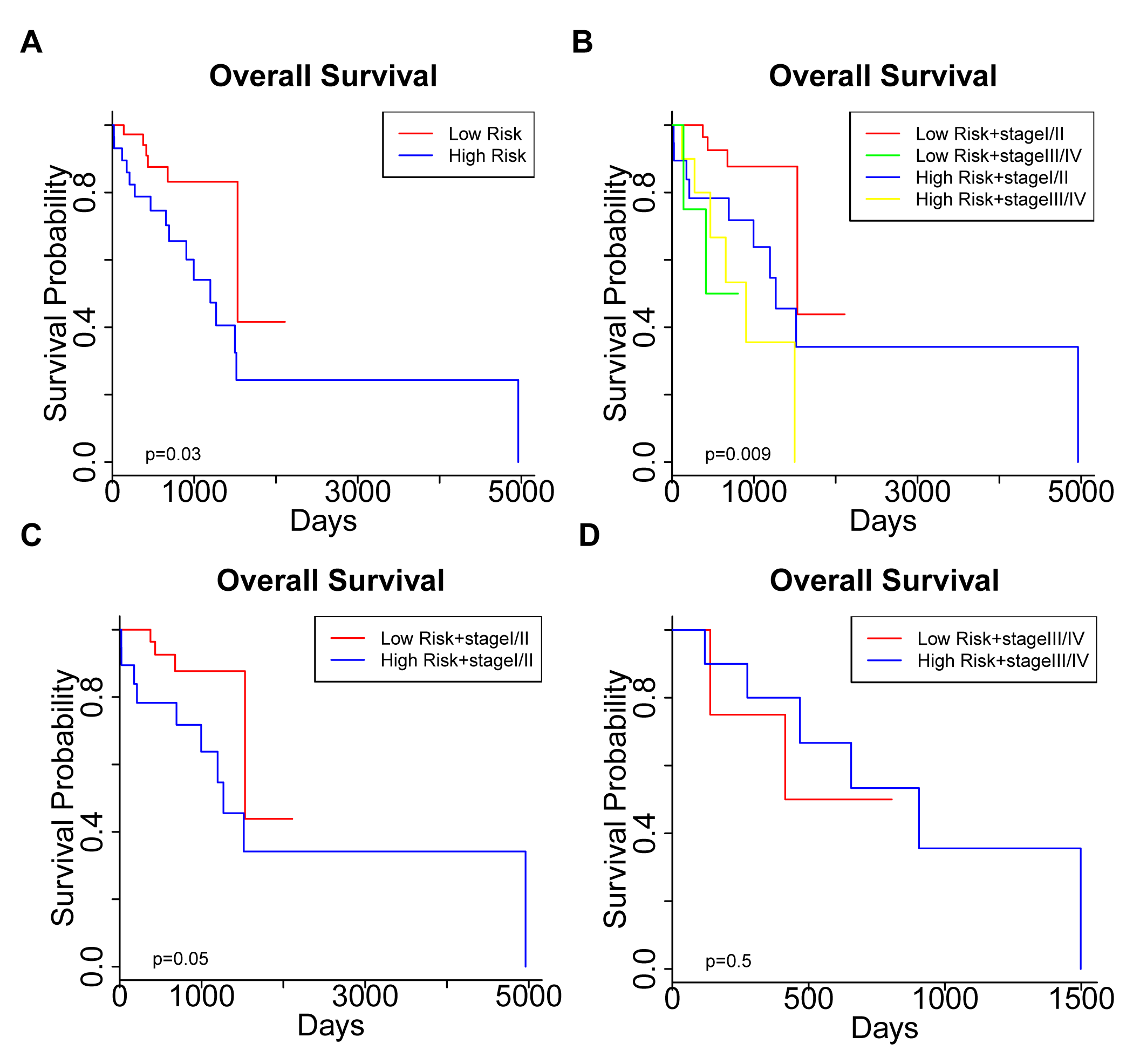

Supplement: Figure S9 — Kaplan Meier curve showing the overall survival (OS) of the non-smokers in our cohort grouped by (A) prognostic score (PS), and (B) PS plus pathological stage. OS of the patients stratified by PS in subgroups with (C) early-stage disease and (D) advanced-stage disease. [file Image_9.TIF]

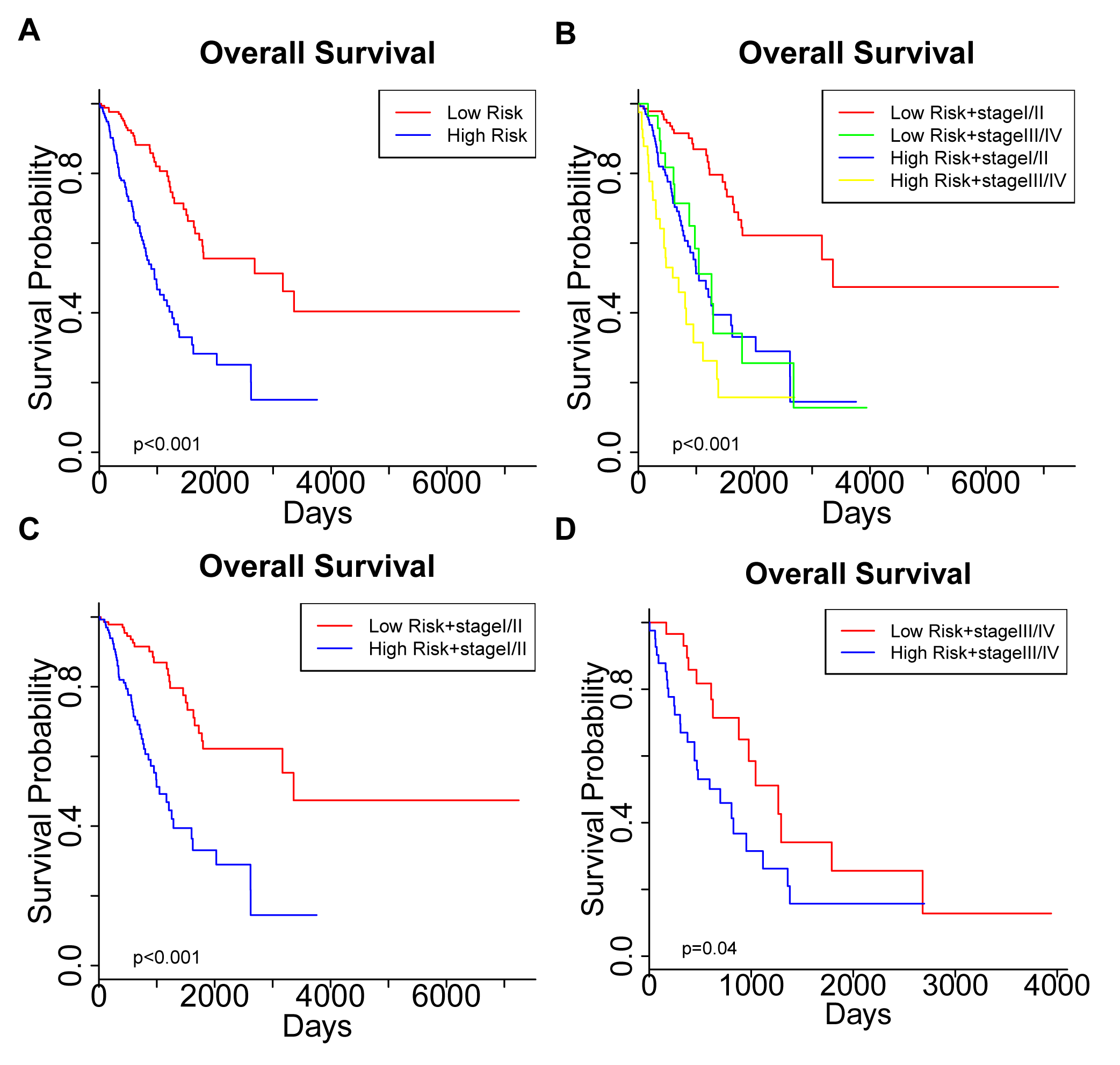

Supplement: Figure S10 — Kaplan Meier curve showing the overall survival (OS) of the smokers in our cohort grouped by (A) prognostic score (PS), and (B) PS plus pathological stage. OS of the patients stratified by PS in subgroups with (C) early-stage disease and (D) advanced-stage disease. [file Image_10.TIF]
