# Supplementary material for: Genome-Wide Analysis of Lung Adenocarcinoma Identifies Novel Prognostic Factors and a Prognostic Score
Source: Front Genet. 2019 May 22;10:493. doi: 10.3389/fgene.2019.00493 (PMC6539224; doi:10.3389/fgene.2019.00493)
Supplement: Table S1 — Transcripts and DNA methylation sites whose expression levels showed significant association with overall survival. [file Table_1.docx]

**Supplementary Tables**

| Supplementary Table 1. Transcripts and DNA methylation sites whose expression levels showed significant association with overall survival | | | | | | | |
| --- | --- | --- | --- | --- | --- | --- | --- |
| Molecular | Characteristics | Coefficient | HR | 95% CI | SE | z value | *p* value |
| miRNA | MIMAT0002890 | 0.239 | 1.27 | 1.037, 1.556 | 0.104 | 2.307 | 0.021 |
|  | MIMAT0004800 | -0.001 | 0.999 | 0.729, 1.371 | 0.161 | -0.003 | 0.997 |
|  | MIMAT0004983 | -0.158 | 0.854 | 0.651, 1.121 | 0.139 | -1.136 | 0.256 |
|  | MIMAT0026472 | 0.021 | 1.021 | 0.821, 1.270 | 0.111 | 0.184 | 0.854 |
|  | MIMAT0019729 | 0.21 | 1.234 | 1.024, 1.488 | 0.095 | 2.204 | 0.028 |
|  | MIMAT0018073 | -0.287 | 0.751 | 0.580, 0.973 | 0.132 | -2.171 | 0.03 |
|  | MIMAT0000269 | 0.038 | 1.039 | 0.763, 1.416 | 0.158 | 0.244 | 0.807 |
|  | MIMAT0000089 | 0.149 | 1.16 | 0.823, 1.637 | 0.175 | 0.848 | 0.397 |
|  | MIMAT0004504 | -0.047 | 0.954 | 0.657, 1.385 | 0.19 | -0.247 | 0.805 |
|  | MIMAT0000243 | 0.099 | 1.104 | 0.754, 1.616 | 0.195 | 0.508 | 0.611 |
|  | MIMAT0000426 | 0.189 | 1.208 | 0.844, 1.729 | 0.183 | 1.031 | 0.302 |
|  | MIMAT0004568 | 0.122 | 1.129 | 0.928, 1.375 | 0.1 | 1.211 | 0.226 |
|  | MIMAT0000258 | 0.012 | 1.012 | 0.694, 1.476 | 0.192 | 0.063 | 0.95 |
|  | MIMAT0004559 | 0.013 | 1.013 | 0.686, 1.496 | 0.199 | 0.065 | 0.948 |
|  | MIMAT0000681 | -0.148 | 0.862 | 0.653, 1.139 | 0.142 | -1.045 | 0.296 |
|  | MIMAT0004549 | -0.119 | 0.888 | 0.617, 1.277 | 0.185 | -0.643 | 0.52 |
|  | MIMAT0004543 | 0.069 | 1.072 | 0.950, 1.210 | 0.062 | 1.126 | 0.26 |
|  | MIMAT0003233 | -0.062 | 0.94 | 0.777,1.136 | 0.097 | -0.642 | 0.521 |
|  | MIMAT0006789 | -0.274 | 0.76 | 0.604, 0.957 | 0.118 | -2.332 | 0.02 |
|  | MIMAT0001620 | -0.036 | 0.965 | 0.826, 1.127 | 0.079 | -0.454 | 0.65 |
|  | MIMAT0003254 | -0.143 | 0.867 | 0.700, 1.073 | 0.109 | -1.311 | 0.19 |
|  | MIMAT0003257 | 0.21 | 1.233 | 0.898, 1.694 | 0.162 | 1.296 | 0.195 |
|  | MIMAT0003247 | 0.22 | 1.246 | 1.079, 1.438 | 0.073 | 2.994 | 0.003 |
|  | MIMAT0004909 | 0.12 | 1.127 | 0.946, 1.342 | 0.089 | 1.342 | 0.18 |
|  | MIMAT0015020 | -0.475 | 0.622 | 0.488, 0.792 | 0.124 | -3.842 | 0 |
|  | MIMAT0004584 | -0.041 | 0.96 | 0.748, 1.231 | 0.127 | -0.325 | 0.745 |
| mRNA | IGFBP1 | 0.074 | 1.077 | 1.005, 1.153 | 0.035 | 2.113 | 0.035 |
|  | TLE1 | 0.323 | 1.382 | 1.071, 1.782 | 0.13 | 2.49 | 0.013 |
|  | PAOX | -0.141 | 0.869 | 0.688, 1.097 | 0.119 | -1.18 | 0.238 |
|  | LASS4 | -0.068 | 0.934 | 0.796, 1.096 | 0.081 | -0.833 | 0.405 |
|  | BZRAP1 | -0.006 | 0.994 | 0.864, 1.145 | 0.072 | -0.077 | 0.939 |
|  | FAHD2B | -0.253 | 0.776 | 0.653, 0.924 | 0.089 | -2.857 | 0.004 |
|  | CLEC17A | -0.187 | 0.83 | 0.736,0.936 | 0.061 | -3.047 | 0.002 |
|  | FAM117A | 0.104 | 1.109 | 0.829, 1.484 | 0.149 | 0.696 | 0.486 |
|  | ZNF540 | 0.024 | 1.025 | 0.877, 1.197 | 0.079 | 0.309 | 0.758 |
|  | DTNBP1 | -0.185 | 0.831 | 0.548, 1.261 | 0.213 | -0.871 | 0.384 |
|  | GNPNAT1 | 0.124 | 1.132 | 0.862, 1.487 | 0.139 | 0.892 | 0.372 |
|  | CYP17A1 | -0.152 | 0.859 | 0.767, 0.963 | 0.058 | -2.613 | 0.009 |
|  | GPR172B | -0.078 | 0.925 | 0.824, 1.039 | 0.059 | -1.319 | 0.187 |
|  | DKK1 | 0.062 | 1.063 | 1.002, 1.129 | 0.03 | 2.027 | 0.043 |
|  | USP4 | -0.339 | 0.712 | 0.459, 1.106 | 0.225 | -1.51 | 0.131 |
| Methylation site | cg27018309 | -1.541 | 0.214 | 0.023, 1.978 | 1.134 | -1.359 | 0.174 |
|  | cg12141052 | 4.161 | 64.129 | 9.691, 424.351 | 0.964 | 4.316 | 0 |
|  | cg12240358 | 0.41 | 1.507 | 0.427, 5.317 | 0.643 | 0.638 | 0.523 |
|  | cg16404170 | 1.512 | 4.535 | 0.765, 26.873 | 0.908 | 1.666 | 0.096 |
|  | cg25229048 | -0.909 | 0.403 | 0.061, 2.673 | 0.966 | -0.942 | 0.346 |
|  | cg20268054 | -0.601 | 0.548 | 0.059, 5.087 | 1.136 | -0.529 | 0.597 |
|  | cg00161124 | 0.565 | 1.76 | 0.572, 5.417 | 0.574 | 0.986 | 0.324 |
|  | cg17490981 | -0.714 | 0.49 | 0.036, 6.705 | 1.335 | -0.534 | 0.593 |
|  | cg26709300 | 1.795 | 6.019 | 1.151, 31.463 | 0.844 | 2.127 | 0.033 |
|  | cg03923535 | -2.162 | 0.115 | 0.017, 0.789 | 0.983 | -2.201 | 0.028 |
|  | cg01105229 | 1.141 | 3.129 | 0.628, 15.581 | 0.819 | 1.393 | 0.164 |
| Abbreviations: HR, hazard ratio; CI, confidence interval. | | | | | | | |

| Supplementary Table 2. Integrated genome-wide prognostic factors in our study | | | | | | | |
| --- | --- | --- | --- | --- | --- | --- | --- |
| Molecular | Name | Coefficient | HR | 95% CI | SE | z value | *p* value |
| miRNA | MIMAT0002890 | 0.306 | 1.358 | 1.127, 1.635 | 0.095 | 3.221 | 0.001 |
|  | MIMAT0000426 | 0.392 | 1.481 | 1.131, 1.938 | 0.137 | 2.857 | 0.004 |
| mRNA | CDADC1 | -0.395 | 0.673 | 0.459, 0.989 | 0.196 | -2.018 | 0.044 |
|  | FAHD2B | -0.3 | 0.741 | 0.622, 0.882 | 0.089 | -3.368 | 0.001 |
|  | BLK | -0.155 | 0.857 | 0.787, 0.933 | 0.043 | -3.565 | <0.001 |
| Methylation site | cg12141052 | 4.281 | 72.315 | 11.235,465.461 | 0.95 | 4.506 | <0.001 |
|  | cg16404170 | 2.962 | 19.346 | 3.463,108.060 | 0.878 | 3.375 | 0.001 |
| Abbreviations: HR, hazard ratio; CI, confidence interval; SE, standard errors of Coefficient; z value: Wald z-statistic value | | | | | | | |
